# The global, regional and national burden of type 2 diabetes mellitus in the past, present and future: a systematic analysis of the Global Burden of Disease Study 2019

**DOI:** 10.3389/fendo.2023.1192629

**Published:** 2023-07-14

**Authors:** Junjun Ye, Yixi Wu, Shuhui Yang, Dan Zhu, Fengwu Chen, Jingxian Chen, Xiaoxia Ji, Kaijian Hou

**Affiliations:** ^1^ Department of Endocrine and Metabolic Diseases, The First Affiliated Hospital of Shantou University Medical College, Shantou, China; ^2^ Shantou University Medical College, Shantou, Guangdong, China; ^3^ Department of Endocrine and Metabolic Diseases, Shantou Central Hospital, Shantou, Guangdong, China; ^4^ Department of Endocrine and Metabolic Diseases, Longhu Hospital, The First Affiliated Hospital of Shantou University Medical College, Shantou, China; ^5^ School of Public Health, Shantou University, Shantou, China

**Keywords:** type 2 diabetes mellitus, epidemiology, global burden of disease, trend, forecast

## Abstract

**Aim:**

To report the global, regional, and national burden of type 2 diabetes mellitus (T2DM) in 2019, assess its trends in the past, and forecast its trends in the future.

**Methods:**

The main data source was the Global Burden of Disease 2019 database. We assessed the changes in T2DM burden from 1990 to 2019 with joinpoint regression analysis. Age-period-cohort analysis was used to forecast the T2DM incidence and mortality rate from 2020 to 2034.

**Results:**

The burden of T2DM has increased from 1990 to 2019 generally. The low-middle socio-demographic index (SDI) region had the highest increase in age-standardized incidence rate (ASIR), age-standardized prevalence rate (ASPR), age-standardized mortality rate (ASMR), and age-standardized disability-adjusted life years (ASDR) due to T2DM. Nationally, the increase in ASIR (r=0.151, p=0.046) and the decrease in ASMR (r=0.355, p<0.001) were positively correlated with SDIs. In 2019, the global ASIR, ASPR, ASMR, ASDR due to T2DM were 259.9 (95% UI 240.3-281.4), 5282.9 (95% UI 4853.6-5752.1), 18.5 (95% UI 17.2-19.7), and 801.5 (95% UI 55477000-79005200) per 100,000 population, respectively. Additionally, the ASIR (r=0.153, p=0.030) and ASPR (r=0.159, p=0.024) of T2DM were positively correlated with SDIs, while ASMR (r=-0.226, p=0.001) and ASDR (r=-0.171, p=0.015) due to T2DM were negatively correlated with SDIs. The ASIR was estimated to increase to 284.42, and ASMR was estimated to increase to 19.1 from 2030 to 2034, per 100,000 population.

**Conclusion:**

Globally, the burden of T2DM has increased in the past and was forecast to continue increasing. Greater investment in T2DM prevention is needed.

## 1 Introduction

Type 2 diabetes mellitus (T2DM), which is characterized by hyperglycemia resulting from progressive loss of adequate insulin production in the setting of insulin resistance, has been identified as a serious global health threat by WHO (1, 2). The International Diabetes Federation estimated that the prevalence of diabetes which was 10.5% in 2021, would increase to 11.3% by 2030 and 12.2% by 2040 (3). T2DM patients have a higher risk of dysfunction and failure of various organs, especially the kidneys, eyes, and nerves, causing increased costs of medical care and decreased life quality (2, 4, 5). In addition, it was observed that T2DM patients had a 15% increased risk of premature death and an approximate 20-year reduced life expectancy (6). The improvement in prevention and prognosis of T2DM has become an urgent medical problem, and studying the epidemiological characteristics of T2DM will contribute to its mitigation.

Previous studies have directly reported the epidemiological data from the database named Global Burden of Disease (GBD) 2019, including the incidence, death rates and disability-adjusted years (DALYs) (7, 8). Additionally, the trends in incidence, death rates and DALYs due to T2DM over the past 30 years have been evaluated by estimated annual percentage changes (EAPCs) (7). However, EAPCs were calculated with the assumption that the trend is linear over the interval, which is contradictory when the data are sparse, giving unreliable results (9). Additionally, previous studies have not assessed the impact of interregional differences in socioeconomic development status on the burden of T2DM and its changes, nor have they forecasted the future trend of T2DM burden. Therefore, more in-depth studies are needed to systematically analysis the global, regional and national burden of T2DM in the past, present and future.

In this study, the burden of T2DM was mainly reflected by its age-standardized incidence rate (ASIR), age-standardized prevalence rate (ASPR), age-standardized mortality rate (ASMR), and age-standardized DALYs due to T2DM (ASDR). The socio-demographic Index (SDI) value was used to evaluate the socio-economic development status of a country or region. We aimed to evaluate the changes of global, regional, and national burden of T2DM from 1990 to 2019, systematically reported the latest epidemiology of T2DM, and assessed the association between them and SDIs. Moreover, the incidence and mortality rate of T2DM from 2020 to 2034 were forecast based on sex and age groups. These results contribute to evidence-based health-policy decision making in different countries and territories.

## 2 Methods

### 2.1 Data source

The GBD project is a comprehensive endeavor that evaluates epidemiological trends and levels related to illnesses and injuries worldwide. In the most recent version of this study, GBD 2019, 369 illnesses and injuries, together with 87 risk variables, were calculated in 21 GBD regions, and 204 countries and territories between 1990 and 2019 (10). In this research, data about the disease burden of T2DM was acquired from GBD 2019, including prevalence, incidence, deaths, and DALYs. In addition, the GBD 2019 global standard population was used to compute the age-standardized rates (ASR) to take into account population and age structure disparities, such as ASPR, ASIR, ASMR, and ASDR. Global population forecasts for 2017-2100 were used to forecast the T2DM disease burden trends.

The SDI value is a composite indicator of the socio-economic development status of a country or region, which is calculated from the overall fertility rate of female under 25-years, the average educational achievement among women at least 15-years of age, and per capita income with a lag in distribution (11). According to the SDI, 204 territories and countries are divided into high SDI regions (SDI>0.81), high-middle SDI regions (0.70≤SDI ≤ 0.81), middle SDI regions (0.61≤SDI ≤ 0.69), low-middle SDI regions (0.46≤SDI ≤ 0.60), and low SDI regions (SDI<0.46) (12). The data mentioned above was from the GBD database (GHDx), which is publicly accessible online at (https://vizhub.healthdata.org/gbd-results ).

### 2.2 Statistical analysis

#### 2.2.1 Descriptive analyses

We conducted the descriptive analyses on the global, regional and national burden of T2DM. Of note, the number of incidence cases, prevalence cases, mortality cases, and DALYs were too large in some regions or countries. To clearly presented those data, we only kept the value of one decimal place of the original data in all tables. When the data was too small in some age groups, it would be presented as “0”. The origin data is publicly accessible at https://vizhub.healthdata.org/gbd-results ). Additionally, we assessed the association between the ASIR, ASPR, ASMR, and ASDR due to T2DM and SDI to explore the factors that influence the national burden of T2DM.

#### 2.2.2 Joinpoint regression analysis

The changes in T2DM disease burden were evaluated using the Joinpoint Regression Program (Version 4.9.1.0.). Joinpoint regression analysis recognizes the best match for inflection points, also known as “joinpoints,” where there is significant change in trends by using a set of permutation tests and multiple comparisons with Bonferroni adjustment. Joinpoint analysis was applied in this study to recognize the independent variable (years) with significant changes in the dependent variable (ASIR, ASPR, ASMR, and ASDR) from 1990 to 2019, together with the size of these changes. We utilized the log-linear model to analyze constant percentage rate change. Up to 5 joinpoints were allowed by employing a Monte Carlo permutation procedure. Each joinpoint reflects a statistically significant (p-value < 0.05) change in trend, with each trend defined by the average annual percentage change (AAPC) and its associated 95% confidence intervals (95% CI). In addition, we assessed the association between the AAPCs of ASIR, ASPR, ASMR, and ASDR caused by T2DM and SDI to explore the factors that influence the changes in the burden of T2DM at the national level.

#### 2.2.3 Age-period-cohort analysis

This study used the age-period-cohort model, a generalized linear model, to analyze the trends in burden of T2DM. The independent variables in the age-period-cohort model are age, period, and cohort, while the dependent variable is the appearance of a particular observable event or phenomenon in the population, which follows a specific probability distribution. Based on the past incidence, mortality rate and the population forecast in GBD 2019, we forecast the incidence and mortality rate of T2DM from 2020 to 2034 using the age-period-cohort model by running the Nordpred package in R. Moreover, to assess the association between incidence, mortality rate and age, we analyzed the trends of incidence rate and mortality rate in different age groups.

The R program was used to perform all statistics (Version 4.2.0). Statistical significance was defined as a p value less than 0.05.

The R program was used to perform all statistics (Version 4.2.0). Statistical significance was defined as a p value less than 0.05.

## 3 Results

### 3.1 Changes in the global, regional, and national burden of T2DM, from 1990 to 2019

Globally, the ASIR of T2DM increased from 184.6 (95% UI 170.9-199.7) per 100,000 population in 1990 to 259.9 (95% UI 240.3-281.4) per 100,000 population in 2019, corresponding to an AAPC of 1.176 (95% CI 1.129-1.224). Similarly, the AAPCs of ASPR, ASMR, ASDR of T2DM, shown in **Table 1**, suggested that ASPR, ASMR, and ASDR due to T2DM were increased around the world.

**Table 1:** The global and regional AAPCs of T2DM ASIR, ASPR, ASMR, and ASDR from 1990 to 2019.

Characteristics	AAPCs			
	ASIR (95% CI)	ASPR (95% CI)	ASMR (95% CI)	ASDR (95% CI)
**Global**	1.176(1.129-1.224)	1.374 (1.319 1.429)	0.365 (0.319 - 0.412)	0.817 (0.738 0.896)
Sociodemographic index				
Low SDI	1.128 (1.066 - 1.191)	1.478 (1.403 - 1.553)	0.178 (0.024 - 0.333)	0.496 (0.444 0.549)
Low-middle SDI	1.433 (1.35 - 1.516)	1.657 (1.446 - 1.868)	0.852 (0.543 - 1.162)	1.116 (0.869 - 1.364)
Middle SDI	1.024 (0.9 1.149)	1.2 (1.143 - 1.258)	0.597 (0.457 - 0.737)	0.815 (0.682 - 0.948)
High-middle SDI	0.938 (0.81 - 1.067)	1.175 (1.144 - 1.207)	-0.142 (-0.233 -0.051)	0.455 (0.381 - 0.528)
High SDI	1.402 (1.263 - 1.54)	1.563 (1.43 - 1.697)	-0.883 (-1.07 -0.694)	0.665 (0.573 - 0.758)
Region				
Andean Latin America	1.532 (1.472 - 1.593)	1.826 (1.801 - 1.85)	0.748 (0.425 - 1.073)	1.016 (0.742 - 1.291)
Australasia	1.458 (1.351 - 1.565)	1.936 (1.8 - 2.072)	-0.754 (-0.977 -0.53)	0.555 (0.415 - 0.696)
Caribbean	0.96 (0.917 - 1.003)	1.215 (1.198 - 1.233)	-0.45 (-0.708 - -0.191)	0.202 (0.066 - 0.338)
Central Asia	2.333 (2.246 - 2.419)	2.328 (2.242 - 2.414)	3,418 (2.866 - 3.972)	2.753 (2.597 - 2.909)
Central Europe	1.348 (1.145 - 1.55)	1,463 (1.36 - 1.567)	0.076 (-0.074 0.225)	0.766 (0.675 0.858)
Central Latin America	0.588 (0.298 - 0.88)	0.98 (0.809 - 1.15)	0.131 (-0.258 - 0.521)	0.399 (0.255 - 0.544)
Central Sub-Saharan Africa	0.892 (0.855 0.93)	1.225 (1.19 - 1.26)	-0.408 (-0.602 - -0.213)	0.042 (-0.105 - 0.189)
East Asia	0.457 (0.159 - 0.756)	0.697 (0.604 - 0.79)	0.183 (0.032 - 0.335)	0.259 (0.042 0.477)
Eastem Europe	1.068 (0.939 - 1.197)	0.971 (0.929 - 1.013)	1.491 (0.606-2.383) -	1.042 (0.821 - 1.263)
Eastern Sub-Saharan Africa	0.465 (0.438 - 0.493)	0.878 (0.846 0.91)	-0.403 (-0.488 - -0.318)	-0.303 (-0.365 -0.241)
High-income Asia Pacific	0.552 (0.413 - 0.691)	0.776 (0.717 - 0.836)	-2.111 (-2.352 -1.868)	-0.006 (-0.071 - 0.06)
High-income North America	1.365 (1.02 - 1.711)	1.456 (1.084 - 1.83)	-0.234 (-0.502 - 0.034)	0.821 (0.596 1.046)
North Africa and Middle East	2.05 (2.015 - 2.085)	2.178 (2.118 - 2.239)	0.074 (-0.088 - 0.237)	0.964 (0.836 - 1.092)
Oceania	1.327 (1.306 - 1.349)	1.792 (1.779 - 1.805)	1.105 (0.984-1.226) -	1.216 (1.138 - 1.293)
South Asia	1.57 (1.49 1.649)	1.769 (1.593 - 1.946)	0.89 (0.277 - 1.507)	1.233 (0.783 - 1.686)
Southeast Asia	1.373 (1.323 - 1.424)	1.624 (1.593 - 1.655)	0.567 (0.486 - 0.648)	0.837 (0.752 - 0.922)
Southern Latin America	1.66 (1.613 - 1.708)	2.06 (1.996 - 2.125)	-0.394 (-0.706 - -0.082)	0.614 (0.478 - 0.75)
Southern Sub-Saharan Africa	1.387 (1.341 - 1.434)	1.65 (1.595 - 1.704)	1.697 (1.161 - 2.235)	1.497 (1.109 - 1.887)
Tropical Latin America	0.364 (0.327 - 0.4)	0.484 (0.44 - 0.527)	-0.419 (-0.64 -0.197)	-0.165 (-0.289 -0.04)
Western Europe	1.326 (1.238 - 1.414)	1.635 (1.582 - 1.689)	-1.494 (-1.589 -1.399)	0.392 (0.281 - 0.504)
Western Sub-Saharan Africa	1.076 (1.058 - 1.095)	1.448 (1.42 1.477)	0.656 (0.558 0.756)	0.779 (0.736 0.821)

All five SDI regions had increased ASIR, among which the low-middle SDI region had the highest AAPC of ASIR, whereas the high-middle SDI region had the lowest. The ASPR, ASMR, ASDR also showed the largest increase in the low-middle SDI region. Of note, the ASMR of T2DM decreased in the high-middle SDI region and high SDI region.

At the regional level, the ASIRs and ASPRs in all GBD regions increased from 1990 to 2019 (**Table 1**). Central Asia had the largest increased burden of T2DM, including ASIR, ASPR, ASMR, and ASDR due to T2DM. Though the ASMRs and ASDRs were increased in most GBD regions, opposite results were observed in some GBD regions. Eight GBD regions had decreased ASMRs, among which Western Europe had the greatest decrease, with an AAPC of -1.494 (95% CI -1.589 to -1.399). Meanwhile, a decrease in ASMRs and ASDRs due to T2DM was found in three GBD regions, including Eastern Sub-Saharan Africa, the High-income Asia Pacific, and Tropical Latin America.

**Figure 1:**
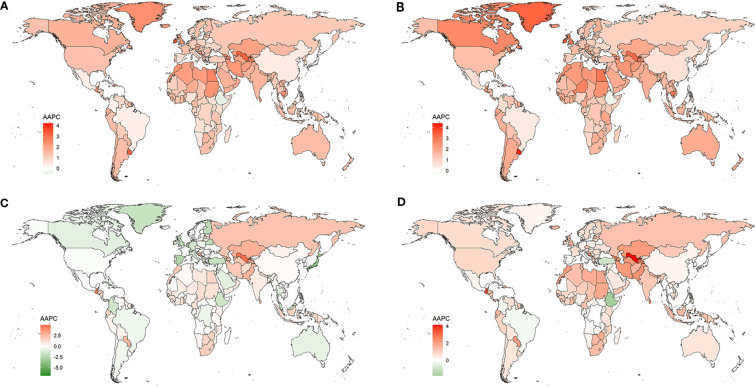
Changes in the ASIR **(A)**, ASPR **(B)**, ASMR **(C)**, ASDR **(D)** of T2DM by countries and territories from 1990 to 2019.

**Figure 2:**
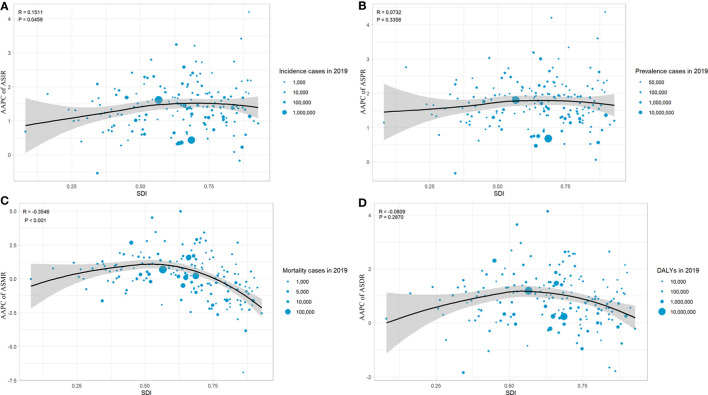
The correlation between the AAPC of ASIR **(A)**, ASPR **(B)**, ASMR **(C)**, ASDR **(D)** of T2DM by countries and territories from 1990 to 2019.

Changes in the burden of T2DM by country and territory from 1990 to 2019 are presented in **Supplementary Table S1**; **Figure 1**. As shown in **Figure 1A**, in almost all countries and territories, the ASIRs of T2DM were increased from 1990 to 2019. The highest increase of ASIR was observed in Luxembourg (AAPC= 4.2 [95% CI 4.153- 4.246]), followed by Ireland. Only Ethiopia and Singapore showed a decreased ASIR, in which AAPC of ASIR was -0.536 (95% CI -0.677 to -0.396) and -0.171 (95%CI -0.374 to 0.033), respectively. Except for Ethiopia, all countries and territories had increased ASPR of T2DM, among which Luxembourg (AAPC= 4.373 [95% CI 4.304- 4.441) was the largest, as shown in **Figure 1B**. Similar to ASIR and ASPR, ASMR (**Figure 1C**) and ASDR (**Figure 1D**) due to T2DM increased from 1990 to 2019 in most of the countries and territories. The largest increase in ASMR and ASDR due to T2DM were in Uzbekistan. Fortunately, in more than 1/3 (84/204) of the countries and territories, the ASMR was decreased, among which Singapore (AAPC= -6.916 [95% CI -7.776 to -6.048]) showed the largest reduction. Additionally, nearly 1/5 (35/204) of the countries and territories had decreased ASDRs due to T2DM, among which Ethiopia (AAPC= -1.836 [95% CI -2 to -1.671]) had the largest decrease, followed by Singapore (AAPC= -1.781 [95% CI -2.375 to -1.184]). Moreover, the correlation between the AAPCs of ASIR, ASPR, ASMR, and ASDR and SDIs was analyzed in this study, and the results are shown in **Figure 2**. The AAPCs of ASIR were positively associated with SDIs (r=0.15, p=0.045, **Figure 2A**). On the contrary, a negative correlation was observed between the AAPCs of ASMR and SDIs (r=-0.35, p<0.01, **Figure 2C**). However, no correlation was found between AAPCs of ASPR and SDIs (r=0.07, p=0.36, **Figure 2B**), nor between AAPCs of ASDR and SDIs (r=-0.08, p=0.29, **Figure 2D**).

### 3.2 The global, regional, and national burden of T2DM in 2019

Globally, as shown in **Table 2**, there were 21,669.9 (95% UI 20,020.9-23,513.5) thousand new cases and a total of 437,906.6 (95% UI 402,043.3-477,018.2) thousand cases of T2DM in 2019. Per 100,000 population, the ASIR was 259.9 (95% UI 240.3-281.4), the ASPR was 5282.9 (95% UI 4853.6-5752.1), and the ASMR was 18.5 (95% UI 17.2-19.7). 66,299.8 (95% UI 55477.0-79005.2) thousand DALYs were caused by T2DM, with an ASDR of 801.5 (95% UI 670.6-954.4) per 100,000 population.

**Table 2:** The global and national incidence cases, ASIR, prevalence cases, ASPR, mortality cases, ASMR, DALYs and ASDR of T2DM in 2019.

Characteristics	Incidence_cases No.×10^3 (95% UI)	ASIR per 100000 NO.(95% UI)	Prevalence_cases No.×10^3 (95% UI)	ASPR per 100000 NO.(95% UI)	Mortality_cases No.×10^3 (95% UI)	ASMR per 100000 NO.(95% UI)	DALYs per 100000 NO.(95% UI)	ASDR per 100000 NO.(95% UI)
**Global**	1669.9 (20020.9-23513.5)	259.9 (240.3-281.4)	437906.6 (402043.3-477018.2)	5282.9 (4853.6-5752.1)	472.9 (1371.9-1565.9)	18.5 (17.2-19.7)	66299.8 (55477-79005.2)	801.5 (670.6-954.4)
Sociodemographic index								
Low SDI	1737 (1581.8-1907.1)	237.2 (217.3-260.6)	28649.2 (25687.8-31899.3)	4690.7 (4226.1-5191.6)	140.3 (127.2-154.7)	31.9 (29-35)	5822.7 (4934-6778.3)	1064.4 (915-1236.7)
Low-middle SDI	4531.3 (4152.5-4954.4)	277.8 (255.3-302.4)	85125.8 (77155.1-93944.5)	5746.9 (5235.1-6306.3)	350.5 (320.2-380.7)	29.1 (26.5-31.5)	14715.5 (12376.8-17318.7)	1049.8 (891.2-1231.4)
Middle SDI	7104.8 (6574.2-7710.3)	261.7 (243.1-283.2)	139200 (127781.1-151612.8)	5330.4 (4909.8-5785.9)	541.5 (503.9-579.6)	23.7 (22-25.5)	23402.7 (19913.2-27520.9)	915.1 (780.4-1075.8)
High-middle SDI	4322.4 (3984.4-4695.9)	232.1 (214.5-251.6)	93989.8 (85771.9-102490.2)	4753.7 (4343-5193.2)	253.1 (232.2-270.7)	12.6 (11.6-13.5)	12282.1 (9912.7-14945.6)	610.2 (492.1-743.7)
High SDI	3959 (3649.7-4283.6)	287.9 (265.5-310.5)	90647.4 (83461.5-97916.7)	5553 (5088-6008.2)	186.1 (167.7-197)	9.1 (8.3-9.5)	10016.1 (7870.4-12530.8)	584.7 (454.6-735)
Region								
Andean Latin America	141.6 (130.7-153.4)	236.3 (218-255.9)	2443.5 (2239.8-2652.1)	4250.7 (3898.4-4602.7)	13.1 (10.9-15.4)	24 (20.1-28.3)	493.6 (408.1-592.3)	873.5 (722.3-1045.3)
Australasia	76.2 (68.5-83.6)	190.5 (172.4-209.3)	1520.3 (1362.2-1688.6)	3368.8 (3006.6-3764.6)	4.8 (4.2-5.2)	8.7 (7.8-9.5)	198.5 (153-250.2)	419.6 (320.7-531.7)
Caribbean	187.5 (173.9-201.6)	366.6 (340.5-393.8)	3848.8 (3562.1-4157.7)	7479.2 (6913.6-8083.3)	18.4 (15.5-21.8)	35.5 (29.9-42.1)	766.9 (633.8-930.7)	1483.1 (1226-1800.5)
Central Asia	243.3 (225.7-262.4)	255.4 (237.9-274.4)	4444.2 (4078-4824.8)	5343 (4931.1-5771.4)	16.6 (14.9-18.5)	23.6 (21.4-26.1)	809.2 (665.8-976.7)	1013.8 (841.9-1219.1)
Central Europe	459.5 (421.8-499.4)	286 (263.2-309.9)	10798 (9798.1-11706.2)	5619.9 (5099.5-6110.2)	26.8 (23.3-30.6)	11.9 (10.4-13.6)	1479.3 (1147-1854.6)	730.2 (559-923.1)
Central Latin America	1072.2 (995.2-1149.3)	418.9 (388.6-448.7)	20824.3 (19192.7-22360)	8505.6 (7837.2-9133.4)	102.7 (90.5-115.6)	44.6 (39.3-50.2)	4206.5 (3578.3-4997.8)	1746.5 (1485.3-2073.9)
Central Sub-Saharan Africa	210 (190.7-230.8)	258 (236-282.2)	3289.3 (2945.4-3654.6)	4955.9 (4481.2-5480.5)	16.9 (13.9-20.7)	38.3 (32.1-46.2)	732.6 (597.4-901.7)	1265.1 (1049.1-1533.2)
East Asia	3894.3 (3585.4-4259.6)	202.1 (186.9-220)	93251.5 (85377.5-101871.9)	4502.3 (4110.8-4930)	182.9 (157.8-208.4)	9.6 (8.3-11)	10164.5 (8071.1-12540.9)	487.6 (387.8-602.7)
Eastern Europe	403.3 (369.2-443.5)	142.6 (131-156)	9148.1 (8294.6-10074.4)	2856.6 (2582.5-3157)	21.6 (19-24.1)	6.1 (5.4-6.8)	1254 (989.7-1562.2)	376 (295.1-468.2)
Eastern Sub-Saharan Africa	412 (376.1-451.7)	176.1 (162.3-191.4)	6067.7 (5427-6771.4)	3052.3 (2760.6-3377.6)	49.1 (43.6-55.7)	36.5 (32.5-40.9)	1748.9 (1499.6-2025.2)	1026.8 (889.8-1182.4)
High-income Asia Pacific	527.5 (481.3-579.1)	194.9 (178.6-212.9)	13222.2 (12034.5-14509.7)	3744.9 (3383.3-4140.2)	21.5 (18.5-23.6)	4.2 (3.7-4.6)	1439.3 (1080.4-1851.5)	383.2 (285.3-495.5)
High-income North America	1665.3 (1542.9-1803.9)	342.3 (317.8-368.3)	38227.8 (35420.2-41150.2)	6725.2 (6220.5-7224.4)	80.3 (73.8-84.1)	12.3 (11.4-12.9)	4392.4 (3527.8-5435)	748.2 (596-931.1)
North Africa and Middle East	2007.3 (1842.5-2191.9)	353.2 (326.1-383.4)	32927.7 (29948-36228)	6753.3 (6170.2-7394.2)	95.4 (84.9-107.4)	25.2 (22.4-28.2)	4806.9 (3927.1-5857.2)	1060.8 (872.1-1279.1)
Oceania	57.2 (53.1-61.8)	506.1 (472.9-542.4)	1021.9 (935.5-1121.7)	11086.1 (10196.7-12087.4)	7.8 (6.3-9.6)	121 (100.2-146.5)	298.1 (244-357)	3703.4 (3060-4399.3)
South Asia	5108.1 (4649.8-5628.1)	299.7 (273.8-329.2)	98137 (88391.9-108844)	6375.2 (5752.2-7067.8)	337.9 (301-379.8)	28.1 (25-31.6)	15119.4 (12449.1-17998.4)	1049.7 (869.3-1244.9)
Southeast Asia	1900.9 (1761.1-2053.7)	273.3 (254-295.6)	31222 (28746.8-33964.8)	4875.1 (4493.5-5285.6)	214.8 (193-236.3)	38.1 (34.1-41.7)	8080 (7008.9-9255.2)	1273.4 (1103.9-1452.4)
Southern Latin America	210.2 (191.7-228.6)	274.6 (249.9-298.4)	4069.9 (3674.7-4445.7)	5031 (4528.4-5494.2)	14.8 (13.7-15.7)	17.4 (16.1-18.5)	593.9 (484.5-720.8)	722.4 (588.2-878.5)
Southern Sub-Saharan Africa	217.9 (202.8-233.7)	326.1 (304.7-349.2)	3389.9 (3098.5-3696.2)	5605.7 (5133.9-6083)	33.9 (31.4-36.6)	68.5 (63.2-73.8)	1063.4 (946.9-1191.2)	1878 (1679.9-2098.8)
Tropical Latin America	677.7 (623.1-740)	268.6 (247.3-292.8)	12302.1 (11179-13438.1)	4979.1 (4525.8-5443.8)	65.3 (59.2-68.9)	28 (25.3-29.6)	2492.5 (2113.1-2920.3)	1020.4 (868-1194.5)
Western Europe	1703.8 (1555.4-1855.1)	276.4 (252.2-301.2)	40769.8 (37016.4-44749.5)	5360.8 (4812.9-5915.5)	93.4 (82.5-99.8)	8.5 (7.6-9.1)	4248.6 (3267.6-5403.5)	515.8 (389.5-663.9)
Western Sub-Saharan Africa	494.2 (451.1-540.4)	197.6 (181.7-214.6)	6980.6 (6275.4-7737.5)	3279.9 (2962.2-3612.2)	55.1 (47.2-63.3)	35.6 (30.8-40.2)	1911.2 (1614-2238.7)	994.8 (851-1148.4)

Among the five SDI regions, the middle SDI region had the most significant number of new cases (**Table 2**) and deaths of T2DM. The highest ASIR was in high SDI region, while the highest ASMR and ASDR due to T2DM were in low SDI region. The low-middle SDI region had the highest ASPR and the largest number of DALYs.

At the GBD regional level, the largest number of new cases of T2DM was in South Asia (**Table 2**). The highest ASIR in 2019 was in Oceania, with the lowest number of cases. Similarly, South Asia had the highest T2DM prevalence, mortality, and DALYs. The highest ASPR, ASMR, and ASDR due to T2DM were also in Oceania.

**Figure 3:**
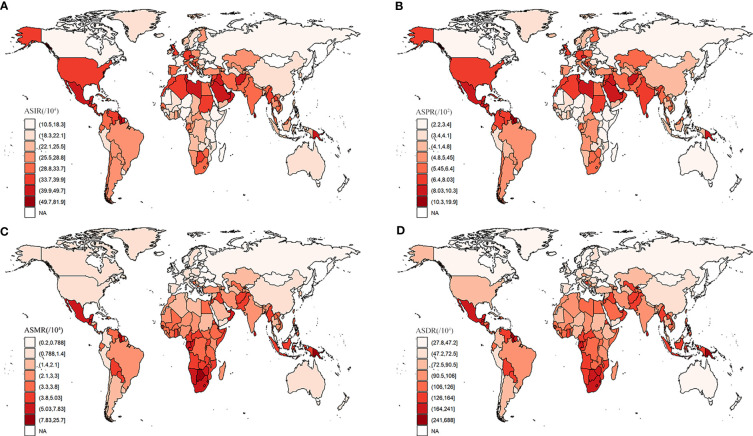
The national burden of T2DM in 2019. **(A)** ASIR, **(B)** ASPR, **(C)** ASMR, **(D)** ASDR.

**Figure 4:**
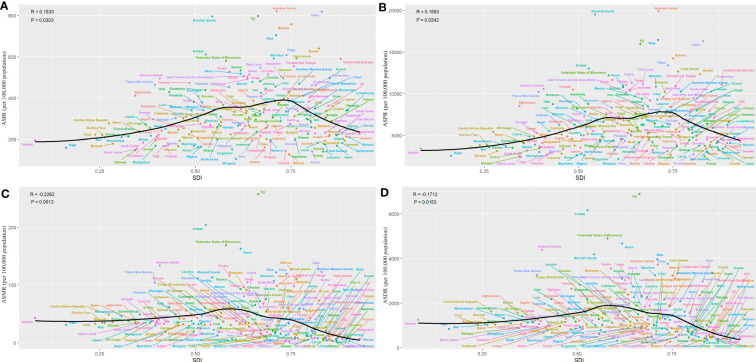
Global T2DM burden of T2DM across 204 countries by SDIs in 2019. **(A)** ASIR, **(B)** ASPR, **(C)** ASMR, **(D)** ASDR.

The national burden of T2DM in 2019 is presented in **Figure 3** and **Supplementary Table S2**. Among 204 countries, India had the greatest number of incidence (4,206,580 [95% UI 3821838.07 – 4647331.08]), following by China and United States of America (**Figure 3A**). The highest ASIRs were in American Samoa (819 [95% UI 763-882] per 100,000 population), Qatar (818 [95% UI 774-869] per 100,000 population), and Fiji (797 [95% UI 764-836] per 100,000 population). In 2019, the highest ASIR (819 per 100,000 population in American Samoa) was 7.8 times higher than the lowest (105 per 100,000 population in Mongolia). Similarly, China and India have the greatest number of prevalence, mortality and DALYs. American Samoa had the highest ASPR in 2019. The highest ASMR and ASDR were seen in Fiji. Moreover, we analyzed the association between the national burden of T2DM and SDI which presented the results in **Figure 4**. The ASIRs were significantly positively associated with SDIs (r=0.15, p=0.03, **Figure 4A**). Similarly, a significant positive association between the ASPRs and SDIs (r=0.16, p=0.02, **Figure 4B**) was observed. On the contrary, a significant negative correlation was observed between the ASMRs (r=-0.23, p<0. 01; **Figure 4C**) and ASDRs (r=-0.17, p=0.02; **Figure 4D**) of T2DM and SDIs in 2019. Notably, in high SDI regions (SDI>0.81), high-middle SDI regions (SDI 0.70-0.81), ASIR, ASPR, ASMR, and ASDR due to T2DM all decreased with the increase of SDIs.

### 3.3 Forecast of global burden of T2DM

**Figure 5:**
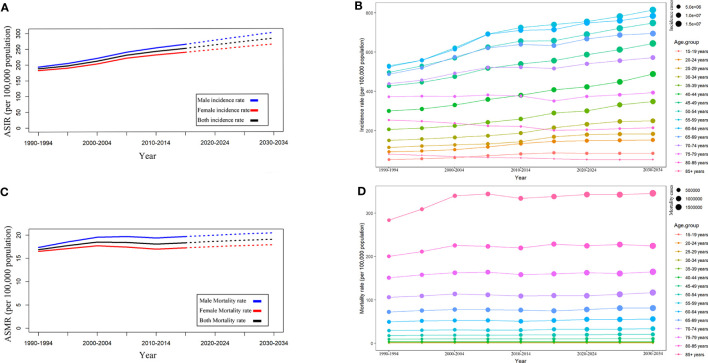
Trends in T2DM incidence and mortality rates by sex and age groups. **(A)** ASIR by sex. **(B)** ASIR by age group. **(C)** ASMR by age groups. **(D)** Mortality by age groups.

Based on the past incidence, mortality rate and the population forecast in GBD 2019, we forecasted the incidence and mortality rate from 2020 to 2034. As shown in **Figure 5**, the ASIR and ASMR of T2DM will still continue to increase in the future. Globally, the ASIR is estimated to increase to, 263.53 per 100,000 population in 2020 to 2024, 274.25 per 100,000 population in 2025 to 2029, and 284.42 per 100,000 population in 2030 to 2034 (**Figure 5A**). The ASMR is estimated to increase to 18.66 per 100,000 population in 2020 to 2024, 18.9 per 100,000 population in 2025 to 2029, and 19.1 per 100,000 population in 2030 to 2034 (**Figure 5C**). Of note, incidence and mortality rates for male have been higher than for female in the past and will remain so in the future.

Moreover, we analyzed the trends in incidence rates (**Figure 5B**) and mortality rates (**Figure 5D**) in different age groups. It was estimated that the incidence rate in most age groups would continue to increase in the future. In 2030-2034, the age group 55-59 will have the highest incidence rate (813.5 per 100,000 population), followed by the age group 60-64 (783.56 per 100,000 population) and age group 50-54 (747.55 per 100,000 population). Different from the incidence rate, the mortality rate of T2DM will remain relatively stable in the future, with the highest mortality rate (345.27 per 100,000 population in 2030-2034) in the 85+age group. Notably, as shown in **Figure 5D**, the mortality rate increased with age.

## 4 Discussion

In this study, we presented a comprehensive picture of the burden and its trends in T2DM in 21 GBD regions and 204 countries and territories from 1990 to 2019, including ASIR, ASPR, ASMR, ASDR. The change trends in T2DM burden were evaluated by AAPCs that were calculated by joinpoint regression analysis. Moreover, we assessed the influence of socio-economic development status on the burden of T2DM and its changes over the past 30 years. Additionally, the incidence and mortality rates of T2DM from 2020 to 2034 were forecast by sex and age groups. All these results help make evidence-based decisions regarding improvements in T2DM prevention and prognosis.

From 1990 to 2019, the global burden of T2DM generally increased. Previous studies have tried to explore the trends in T2DM with percentage change and the EAPCs (7, 8). EAPCs were calculated assuming the trend is linear over the interval, which is concerning when the data are sparse, causing inconsistent results. In this study, we used AAPCs, which considered all inflection points from 1990 to 2019, to assess the trends in T2DM burden from 1990 to 2019. When the data are sparse, AAPC is especially reliable for characterizing small segments based on joinpoint models fitted over considerably longer periods (9). Therefore, compared to the previous studies, this study found a more accurate result which suggested worsening changes in T2DM burden. For example, the ASIR and ASDR were decreased while evaluating by EAPC in previous study, but opposite results was found in this study. The largest increased burden of T2DM from 1990 to 2019, including ASIR, ASPR, ASMR, and ASDR, was found in the low-middle SDI region. Fortunately, the high-middle and high SDI regions had a decreased ASMR. Similarly, at the national level, though the T2DM burden generally increased in most countries and territories, an opposite result was shown in some countries and territories. The ASMR decreased in over 1/3 of countries and territories, and the ASDR decreased in nearly 1/5 of countries and territories. Further analysis suggested that the ASIR of T2DM was positively corrected with SDIs, while the ASMR of T2DM was negatively associated with SDIs, but no association was observed between both ASPR or ASDR and SDIs. However, possibly due to the difference in database or observation periods, a multi-country analysis found that the rate of diagnosis of diabetes was stabilizing or declining from 1995 to 2018 in many high-income countries (13).

In the past 30 years, the global economy has flourished, especially in developing countries, and significant changes in food, life, and work patterns have accompanied this change. Lightly processed foods have been replaced by heavily processed foods high in calories, saturated fatty acids, sugar, and salt (14–16). Rapid urbanization and industrialization have caused enormous environmental pollution, especially in the air (17). Additionally, the work pattern has become more sedentary, causing reduced activity levels (17, 18). These factors are considered to play important roles in the development of T2DM. Therefore, it was unsurprising that the ASIR and ASPR increased in most countries, especially in low-middle SDI countries. Unfortunately, it seems that healthcare systems in low-SDI countries have not kept pace with their economic development, causing more disease burden from chronic diseases, including T2DM (19–21). In addition, existing medications are not effective enough to control blood glucose, and adherence in patients with T2DM is inadequate (22, 23). The prognosis for T2DM remains poor due to these factors. Therefore, there is an urgent need to improve the medical systems to reduce the burden of T2DM globally, especially in low-income countries (24).

The burden of T2DM varies between countries and territories. In 2019, the highest ASIR was in the high SDI region. Further analysis also suggested that the ASIR of T2DM was positively associated with SDIs value. The highest ASPR was observed in the low-middle SDI region, but a positive correlation was observed between ASPR and SDIs upon further analysis. For reasons, obesity rates are generally high in high SDI countries, which is closely related to the development of T2DM (25, 26). Additionally, premium medical and health resources were more readily available in high SDI countries, causing a higher early diagnosis rate of T2DM (27, 28). Therefore, though low SDI countries had a lower T2DM incidence rate compared to high SDI countries, they should pay more attention to the early diagnosis of T2DM that may lead to a better prognosis (29, 30). From the perspective of the prognosis of T2DM, the low-middle SDI region has the highest ASMR and ASDR due to T2DM. It was not surprising that further analysis suggested negative associations between both ASMR and ASDR and SDIs. The prognosis of T2DM tended to be worse in low SDI countries, possibly due to limited medical resources and technology (28). In addition, there were also significant differences between and within low-income and high-income countries in terms of diagnosis and treatment of diabetes (31, 32). Approximately 53% of global healthcare spending on diabetes was in the United States. In comparison, less than 1% of that was spent in India, which has one of the highest numbers of diabetes patients, and only 0.3% of that was spent in an African region consisting of 18 countries (33). Moreover, a previous multicenter study on T2DM patients found that only 20-30% of T2DM patients can control their HbAlc level within the recommended limits (22). Therefore, improving the prognosis of T2DM is still an essential task in all countries.

In this study, the incidence rates and mortality rates of T2DM from 1990 to 2019 were analyzed by gender and age group, and those from 2020 to 2034 were forecast with the Nordpred package in R. Worryingly, the incidence rate was forecast to keep increasing from 2019 to 2034. Previous studies only forecasted the incidence rate of all types of diabetes, so the results of previous studies and this study cannot be compared directly (15). However, all results show that the incidence rate will keep increasing. Overall, T2DM incidence and mortality rates for men have been higher than for women and will remain so. The sex distribution of T2DM is strongly associated with the difference in physiology and metabolism, lifestyle, education attainment, cultural factors and socioeconomic status between the genders (34, 35). Previous studies have discovered that different countries have varying burden disparities between genders (36–38). Overall, females are more concerned about the treatment of T2DM and have higher treatment compliance, which may contribute to a better prognosis of T2DM (39). Moreover, differences in incidence and mortality rate by age group also play important roles in decision-making for prevention and prognosis improvement policies for T2DM. According to our forecast, the five age groups of 55-59, 60-64, 50-54, 45-49, and 40-44 years will have the highest incidence rates in that order. Fortunately, the mortality rate of T2DM will remain relatively stable as forecasted. The mortality rate of T2DM increased with age in the past and will remain so in the future, possibly due to old adults having more underlying diseases and polypharmacy. A systematic review suggested a positive correlation between polypharmacy in old adults and the risk of hypoglycemia, poor glycemic control, incident falls, hospitalization, syncope, and death (40). Of note, Covid-19 (SARS-CoV-2) has significantly impacted the health of all humans since 2019. The human pancreas is one of the target organs of covid-19, and covid-19 may damage beta cells by triggering pro-inflammatory cytokines, which may cause hyperglycemia and insulin resistance (41–43). Moreover, a retrospective cohort analysis estimated that covid-19 patients presented an increased T2DM incidence compared to other acute upper respiratory tract virus infections (44). Therefore, the future morbidity and mortality of T2DM may be even higher than our estimates suggest.

In summary, the burden of T2DM increased globally from 1990 to 2019. The low-middle SDI region had the highest increased T2DM burden. In 2019, the highest ASIR of T2DM was observed in the high SDI regions, whereas the highest ASPR, ASMR, and ASDR due to T2DM were observed in the low-middle regions. As forecast, the global incidence rates and mortality rates of T2DM will keep increasing. The 40-70 age group will have the highest incidence rate, while the 60-85+ age group will have the highest mortality rate of T2DM from 2020 to 2034. Therefore, a more robust investment in T2DM prevention is needed in high SDI regions and in the 40-70 age group, while prognosis improvement of T2DM is needed more in low SDI regions and in the 60-85+ age group to prevent an increased burden of T2DM. However, there were some limitations in this study. Due to differences in the speed of economic development and the foundation of health systems in different countries, GBD database was difficult to adopt a unified standard to accurately determine the burden of disease. Stronger leadership and political support are needed for continuous monitoring and evaluation of prevention programs. The establishment of national or subnational diabetes registries can provide comprehensive data on the epidemiology, risk factors, and outcomes of diabetes at both individual and population levels. Moreover, the registry can facilitate the integration of diabetes care across healthcare sectors and enhance collaboration between researchers, clinicians, policymakers, and patients.

## Data availability statement

Publicly available datasets were analyzed in this study. This data can be found here: https://vizhub.healthdata.org/gbd-results.

## Author contributions

JJY and YXW are responsible for the design and manuscript writing. JJY, YXW, SHY, DZ, JXC, and XXJ are responsible for editing the structure of the article, obtaining the documents, and further arranging the manuscripts. KJH is responsible for the supervision, review, and final editing of the manuscript.
