# Supplementary material for: The global, regional and national burden of type 2 diabetes mellitus in the past, present and future: a systematic analysis of the Global Burden of Disease Study 2019
Source: Front Endocrinol (Lausanne). 2023 Jul 14;14:1192629. doi: 10.3389/fendo.2023.1192629 (PMC10376703; doi:10.3389/fendo.2023.1192629)
Supplement: Supplementary file 1 [file Table_1.docx]

Supplementary Table 1 | Continued

Supplementary Table 1 | Continued

Supplementary Table 1 | Continued

Supplementary Table 1. The national AAPCs of T2DM ASIR, ASPR, ASMR, and ASDR from 1990 to 2019.
