# Supplementary material for: The global, regional and national burden of type 2 diabetes mellitus in the past, present and future: a systematic analysis of the Global Burden of Disease Study 2019
Source: Front Endocrinol (Lausanne). 2023 Jul 14;14:1192629. doi: 10.3389/fendo.2023.1192629 (PMC10376703; doi:10.3389/fendo.2023.1192629)
Supplement: Supplementary file 2 [file Table_2.docx]

Supplementary Table 2 | Continued

Supplementary Table 2 | Continued

Supplementary Table 2 | Continued

Supplementary Table 2 | Continued

Supplementary Table 2 | Continued

Supplementary Table 2 | Continued

Supplementary Table 2 | Continued

Supplementary Table 2. The national incidence cases, ASIR, prevalence cases, ASPR, mortality cases, ASMR, DALYs and ASDR of T2DM in 2019
